# Second stage versus first stage intrapartum cesarean delivery and adverse maternal and perinatal outcomes in Southern Ethiopia

**DOI:** 10.1186/s12884-026-09216-9

**Published:** 2026-05-08

**Authors:** Ephrem Kedir, Temesgen Teklu Alemayehu, Mihiretu Tesfamriam Goshu

**Affiliations:** 1 Department of Obstetrics and Gynecology, School of Medicine and Health Sciences, Hawassa University, Hawassa, Ethiopia; 2 Department of Obstetrics and Gynecology, Besheno Primary Hospital, Halaba, Ethiopia

**Keywords:** Cesarean section, Second stage labor, First stage labor, Maternal adverse outcome, Perinatal adverse outcome

## Abstract

**Background:**

Cesarean section performed during the second stage of labor is technically challenging and associated with higher maternal and neonatal morbidity. This study aimed to assess adverse maternal and perinatal outcomes for women who have a cesarean section performed during the first and second stages of labor at Hawassa University Comprehensive Specialized Hospital.

**Methods:**

An institution-based comparative cross-sectional study was conducted from May to November 2024 among 278 term women who underwent intrapartum cesarean delivery. Among these, 193(69.4%) had first-stage cesarean delivery, and 85(30.6%) had second-stage cesarean section. Data were collected using structured questionnaires and medical record review. Multivariable logistic regression analysis was performed to identify factors associated with adverse maternal and perinatal outcomes.

**Results:**

During the study period, there were 1434 deliveries, of which 716 were cesarean sections, yielding a cesarean delivery rate of 49.9%. The overall rate of adverse maternal and perinatal outcomes was 24.5% and 29.1%, respectively. Compared with the first-stage cesarean section, second-stage cesarean delivery is associated with increased odds of adverse maternal outcomes (AOR = 4.20, 95% CI: 1.95–9.05) and adverse perinatal outcomes (AOR = 3.05, 95% CI: 1.65–5.65).

**Conclusion:**

Cesarean section performed during the second stage of labor was significantly associated with adverse maternal and perinatal outcomes. During the second stage of cesarean delivery, the obstetric team’s preparedness for potential intraoperative complications and immediate neonatal resuscitation should be strengthened.

## Introduction

Cesarean Section (CS) is a surgical procedure used to deliver a fetus when vaginal delivery is unsafe for the mother or fetus after 28 weeks of gestation [1]. Even though it is a life-saving intervention, the World Health Organization (WHO) recommends limiting the population-level cesarean delivery rates within 10–15%, beyond which the protective effect of cesarean intervention on maternal and neonatal outcomes diminishes [2]. Despite this recommendation, more than one in five (21%) births worldwide currently are performed via CS, and projections show that by 2030, over 30% of deliveries will take place by cesarean Section  [3].

Because CS is associated with both short-term and long-term maternal and neonatal complications, the rising rate of cesarean delivery has raised concerns, and these risks are particularly noticeable in low- and middle-income countries (LMICs) [4]. Cesarean delivery can be performed in the first or second stage of labor. Second-stage CS, which is defined as CS performed after full cervical dilatation, presents additional procedural difficulties due to deep engagement of the fetal head in the maternal pelvis [5, 6]. These challenges increase the risk of uterine extension, excessive blood loss, bladder injury, and longer operation time, as well as neonatal complications, poor Apgar score, neonatal intensive care unit (NICU) admission, fetal iatrogenic injury, neonatal sepsis, and neonatal death [7, 8].

In Ethiopia, the cesarean delivery rate has increased over the past few years, with variation across regions. According to the Ethiopia Demographic and Health Survey (EDHS) 2019, results showed that 5% of live births in the five years before the survey were delivered by cesarean section, highlighting low population-level coverage [9]. In comparison, studies conducted at various higher institutions and referral hospitals show a higher CS rate. A systematic review conducted in Ethiopia estimated an overall cesarean section prevalence of 29.5%, while studies specific to facilities in urban referral areas report even higher figures [10]. For instance, a 2017 study at a public and private hospital in Hawassa City found that 49.3% of deliveries were cesarean, and another study from Addis Ababa reports a 38.3% cesarean delivery rate, reflecting the concentration of complicated and high-risk cases at higher-level facilities [11, 12]. Common indications in this setting include cephalopelvic disproportion, obstructed labor, fetal distress, and abnormal presentation, which predispose to second-stage cesarean Section  [11–13].

Despite the growing trend of cesarean section in Ethiopia, literature is lacking in the Sidama region that compares maternal and perinatal outcomes between first- and second-stage cesarean delivery, and most studies have been conducted in settings with different healthcare infrastructures and resource availability. To help practitioners in identifying existing clinical gaps and applying findings to improve clinical practice, it is essential to examine the correlations between cesarean sections performed in the first and second stages of labor and subsequent adverse maternal and newborn outcomes. Understanding these associations will provide valuable insights that could inform clinical decision-making and ultimately improve fetomaternal outcomes in similar low-resource settings. Therefore, generating context-specific evidence is essential to better understand the magnitude and determinants of adverse maternal and perinatal outcomes in these settings. This study aimed to assess adverse maternal and perinatal outcomes among women undergoing cesarean section in the first and second stages of labor at Hawassa University Comprehensive Specialized Hospital (HUCSH).

## Methods

### Study design and participants

An institution-based comparative cross-sectional study was conducted from May 1 to November 30, 2024, at HUCSH in Hawassa town, Sidama National Regional State, Southern Ethiopia. The hospital provides obstetric services, averaging 4200 deliveries per year, and serves a population of approximately 18 million people. The obstetric care is provided by a team comprising consultant obstetricians, obstetrics and gynecology residents, and midwifery nurses. The diagnosis of labor abnormalities and indications for cesarean section are primarily made by junior residents and are confirmed by senior residents or consultant obstetricians. Cesarean sections are performed by senior residents or consultant obstetricians, often with the assistance of junior residents or interns. The study was conducted among term pregnant women who underwent intrapartum cesarean delivery at HUCSH during the study period and excluded mothers with Intrauterine Fetal Death (IUFD), lethal congenital anomalies, multifetal gestation, and previous cesarean section. Before data collection, ethical clearance was obtained from the Institutional Review Board of Hawassa University College of Medicine and Health Sciences (Ref.No: IRB/328/16).

### Sample size determination and sampling technique

**Fig. 1:**
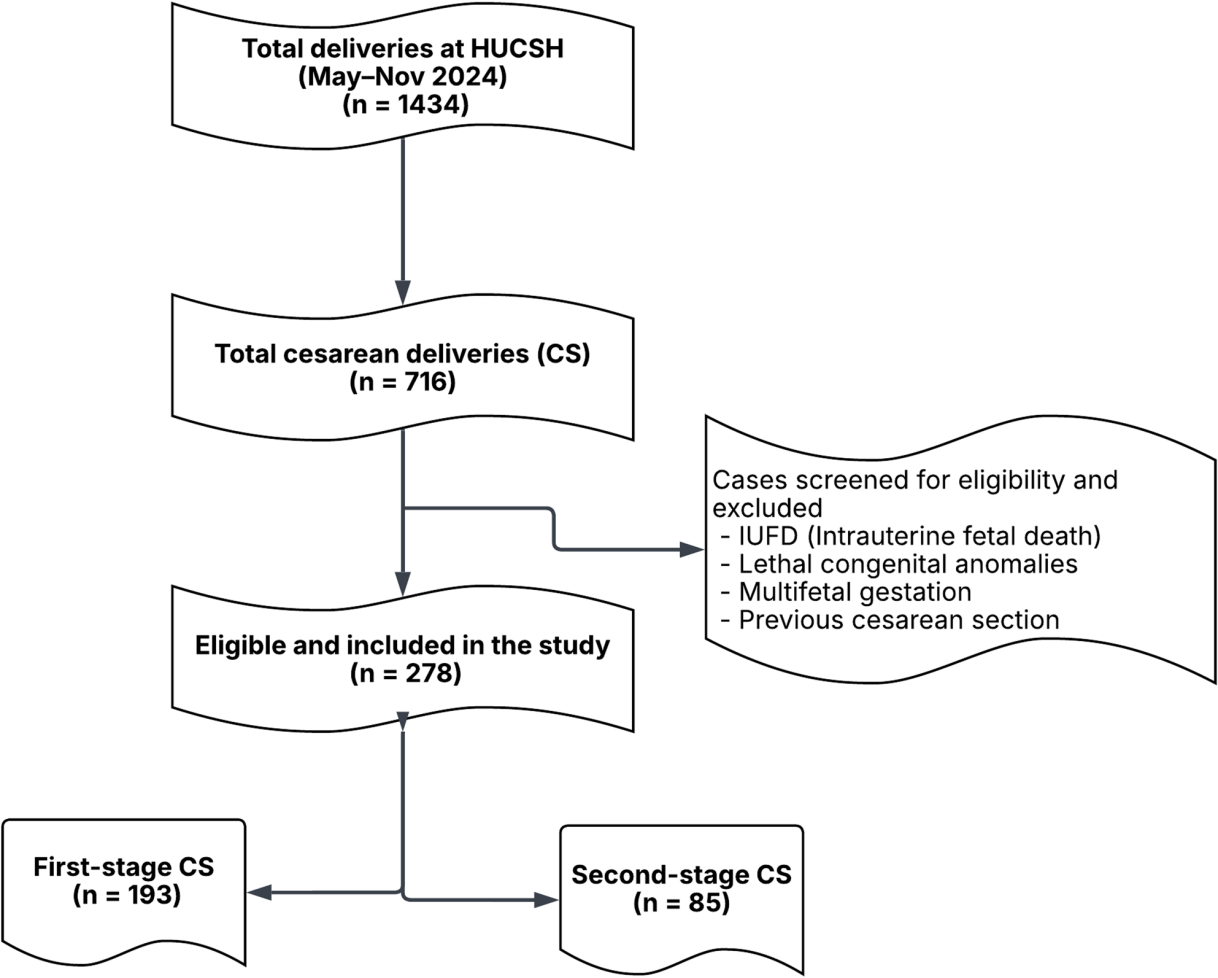
Flowchart of participant selection for the study

The sample size was determined using OpenEpi version 3 to compare two proportions with unequal group sizes. Based on study conducted in Wolkite University Hospital, Ethiopia, reported adverse maternal outcome rates of 12.2% in first-stage cesarean section and 34.1% in second-stage cesarean section (OR = 3.7), a minimum of 198 study participants were required to achieve 80% power at a 95% CI, accounting for the expected population distribution of cesarean section by stage (85% first-stage, 15% second-stage) [14]. This study used oversampling by including all eligible second-stage cases during the study period (*n* = 85) to ensure adequate representation of the second-stage group. First-stage cases were sampled by enrolling the next two eligible first-stage cases following each second-stage case to maintain an approximate 1:2 ratio, giving 193 first-stage cases and a final sample of 278 participants. The participant selection process is illustrated in Fig. 1.

### Variables

After reviewing relevant literature, a structured interviewer-administered questionnaire and a medical record review checklist were developed to collect data. The questionnaire assessed sociodemographic characteristics, obstetric characteristics, and clinical presentations, while maternal and fetal outcome parameters were extracted from medical records. The structured questionnaire was completed after the woman delivered and before she or the neonate was discharged from the hospital. The primary outcomes of interest were adverse maternal and neonatal outcomes. The data collection tool was adapted from a previously published study conducted at Jimma Medical Center, Ethiopia, and modified to suit the objectives and context of the current study, and is provided in the supplementary material (Appendix 1) [8].

### Operational definitions

Labor: initiation of regular, painful uterine contractions that result in effacement, dilatation, and delivery of a fetus [15].

First stage of labor (FSOL): from onset of labor to full dilatation of cervix (10 cm of cervical dilatation) [15].

Second stage of labor (SSOL): from full dilatation of the cervix to delivery of the fetus [15].

Term pregnancy: delivery within gestational age 37 to 42 complete weeks [16].

Adverse maternal outcome: maternal complications, which include one or more of the following: estimated blood loss more than 1000 ml, unintentional uterine incision extension, uterine atony, blood transfusion, puerperal sepsis, and prolonged hospital stay for more than 5 days [14].

Adverse perinatal outcome: one or more of the following neonatal complications: fifth-minute Apgar score < 7, need for NICU admission, and neonatal death [14].

Extension of uterine incision: any tear on the uterus that requires additional layer of repair [8].

Arrest of descent: failure of the fetal presenting part to descend despite full cervical dilatation and adequate uterine contractions during the second stage of labor.

### Data analysis

Data was entered and analyzed using the Statistical Package for the Social Sciences (SPSS), version 26. Descriptive statistics summarized participants’ socio-demographic characteristics, maternal obstetric and labor factors, and indications for cesarean section. Categorical variables, including maternal age category, residence, educational status, indication for cesarean section, birth weight category, maternal and perinatal outcomes, gravidity, antenatal care attendance, onset of labor, and fetal station, were compared using Chi-square or Fisher’s exact test, as appropriate. Continuous variables, including maternal age (in years), operation time (in minutes), length of hospital stay (in days), and neonatal Apgar score at 5 min, and estimated blood loss (in milliliters) were compared using the independent t-test. Binary logistic regression analyses were performed to identify candidate variables associated with the outcome of interest. Variables with clinical relevance and a p-value ≤ 0.25 in the binary logistic regression were subsequently included in a multivariable logistic regression model. Model fit was assessed using the Hosmer– Lemeshow test. The strength of associations was reported using adjusted odds ratios (AORs) with corresponding 95% confidence intervals (CIs). Statistical significance was determined at a two-sided alpha level of 0.05.

## Result

### Socio-demographic characteristics of the respondents

During the study period, there were 1434 deliveries, of which 716 were cesarean sections, yielding a cesarean delivery rate of 49.9%. The study included 278 participants who gave birth by cesarean section at HUCSH, of whom 193 (69.4%) had cesarean births carried out in the first stage of labor, and 85 (30.6%) underwent cesarean birth in the second stage of labor. Maternal age, educational status, and marital status did not differ significantly between women undergoing first-stage and second-stage cesarean delivery. In contrast, a higher proportion of women from rural areas underwent second-stage cesarean delivery compared with first-stage cesarean delivery (31.8% vs. 9.8%, *p* < 0.001) (Table 1).

**Table 1:** Socio-demographic characteristics of women undergoing cesarean delivery at Hawassa University Comprehensive Specialized Hospital, 2024 (*N* = 278)

Characteristic	First-stage CS (*n* = 193)	Second-stage CS (*n* = 85)	*p*-value
Age category, *n* (%)			0.653
<20	6 (3.1)	1 (1.2)	
20–24	71 (36.8)	27 (31.8)	
25–29	75 (38.9)	40 (47.1)	
30–35	31 (16.1)	12 (14.1)	
>35	10 (5.2)	5 (5.9)	
Educational Status, *n* (%)			0.440
unable to read and write	36(18.6)	20(23.8)	
More than primary education^#^	157(81.4)	65(76.2)	
Residence, *n* (%)			< 0.001
Urban	174 (90.2)	58 (68.2)	
Rural	19 (9.8)	27 (31.8)	
Marital status, *n* (%)			0.64
Married	190 (98.4)	83 (97.6)	
Not Married*	3 (1.6)	2 (2.4)	

CS: Cesarean section

*Includes single and widowed women

#Includes secondary and college education

### Maternal obstetric characteristics and indications for cesarean delivery

Women who had a cesarean section in the second stage of labor had more newborns with macrosomia than those in the first-stage group (*p* < 0.041). In contrast, labor induction was more common in the first-stage group than in the second stage (22.8% vs. 7.1%, *p* < 0.002). There was no significant difference between the first- and second-stage groups in ANC follow-up or gravidity. Regarding the indication for cesarean delivery, a significant difference was observed between the two groups: cephalopelvic disproportion and obstructed labor were more common in the second-stage group (52.9% vs. 1.6%, *p* < 0.001) and (27.1% vs. 0%, *p* < 0.001), respectively. In contrast, a non-reassuring fetal heart rate pattern (NRFHRP) was the leading indication in the first stage group (61.7% vs. 11.7%, *p* < 0.001) (Table 2).

**Table 2:** Maternal obstetric characteristics and indications for cesarean delivery among women who underwent cesarean delivery (*N* = 278)

Variable	Category	First stage of labor (FSOL) *n* = 193	Second stage of labor (SSOL) *n* = 85	*p*-value
ANC attendance	No (0 visits)	3 (1.6%)	1 (1.2%)	0.762
	Yes (≥ 1 visit)	190 (98.4%)	84 (98.8%)	
Gravidity	Multigravida	98 (50.8%)	46 (54.1%)	0.603
	Primigravida	95 (49.2%)	39 (45.9%)	
Fetal station at CS	−2	16 (8.3%)	2 (2.4%)	< 0.001
	−1	76 (39.4%)	9 (10.6%)	
	0	84 (43.5%)	32 (37.6%)	
	+ 1	14 (7.3%)	36 (42.4%)	
	+ 2	3 (1.6%)	6 (7.1%)	
Onset of labor	Induced	44 (22.8%)	6 (7.1%)	0.002
	Spontaneous	149 (77.2%)	79 (92.9%)	
Birth weight	≥ 4000 g	17 (8.8%)	15 (17.6%)	0.041
	< 4000 g	176 (91.2%)	70 (82.4%)	
Indication for cesarean delivery	Arrest of descent	0 (0.0%)	2 (2.4%)	0.103
	Arrest/protracted dilatation	13 (6.7%)	2 (2.4%)	0.229
	CPD	3 (1.6%)	45 (52.9%)	< 0.001
	MSAF	47 (24.3%)	2 (2.4%)	< 0.001
	NRFHRP	119 (61.7%)	10 (11.7%)	< 0.001
	Obstructed labor	0 (0.0%)	23 (27.1%)	< 0.001
	Other	11 (5.7%)	1 (1.2%)	0.004

*Abbreviations*: *ANC* antenatal care, *CS* cesarean section, *CPD* cephalopelvic disproportion, *MSAF* meconium-stained amniotic fluid, *NRFHRP* non-reassuring fetal heart rate pattern, *FSOL* first stage of labor, *SSOL* second stage of labor

### Perioperative adverse maternal and perinatal outcomes

Maternal and neonatal perioperative adverse outcomes varied by the stage of labor at cesarean delivery (Table 3). In this study, the overall rate of adverse maternal outcome was 68(24.5%), with a significantly higher proportion among those undergoing second-stage cesarean sections compared to first-stage cesarean Sect.  (50.6% vs. 13%). Similarly, adverse perinatal outcomes were observed in 81 (29.1%) neonates, occurring more frequently following second-stage cesarean delivery than first-stage cesarean delivery (49.4% vs. 20.2%). Cesarean deliveries performed during the second stage of labor were associated with longer operative times than in first-stage cesarean deliveries, with a higher proportion lasting more than 50 min (63.5% vs. 36.8%). After second-stage cesarean delivery, maternal complications such as uterine incision extension, uterine atony, blood transfusion, and puerperal sepsis occurred more frequently (all *p* < 0.05). Additionally, women undergoing a second-stage cesarean section were more likely to stay in the hospital for more than five days (20.0% vs. 7.8%).

**Table 3:** Maternal and neonatal adverse outcomes by stage of labor at cesarean delivery (*N* = 278)

Outcome	First-stage CS (*n* = 193)	Second-stage CS (*n* = 85)	*p*-value
Adverse maternal outcome	25(13%)	43(50.6%)	**< 0.001**
EBL ≥ 1000 mL	3 (1.6%)	3 (3.5%)	0.380
Uterine incision extension	2 (1.0%)	6 (7.1%)	**0.011**
Uterine atony	2 (1.0%)	7 (8.2%)	**0.005**
Blood transfusion	1 (0.5%)	5 (5.9%)	**0.025**
Puerperal sepsis	2 (1.0%)	5 (5.9%)	**0.025**
Hospital stay > 5 days	15 (7.8%)	17 (20.0%)	**0.004**
Operation time > 50 min	71 (36.8%)	54 (63.5%)	**< 0.001**
Mean operation time (min)	44.13 ± 13.4	55.52 ± 15.1	0.006
Mean EBL (mL)	392.2 ± 202.7	479.8 ± 178.5	**0.002**
Mean hospital stay (days)	3.53 ± 3.68	4.06 ± 2.35	**0.192**
Adverse perinatal outcome	39 (20.2%)	42 (49.4%)	**< 0.001**
5-minute Apgar score < 7	7 (3.6%)	12 (14.1%)	**0.001**
NICU admission	26 (13.5%)	28 (32.9%)	**< 0.001**
Neonatal death	6 (3.1%)	2 (2.4%)	1.000
Mean Apgar score at 5 min	8.88 ± 0.95	8.42 ± 1.66	**0.009**

*Abbreviations*: *CS* cesarean section, *EBL* estimated blood loss, *NICU* neonatal intensive care unit

Bold value indicates statistical significance (*p* < 0.05)

The mean 5-minute Apgar score was significantly lower in the second-stage group (*p* < 0.009), and neonates delivered by second-stage cesarean delivery were more likely to have a 5-minute Apgar score < 7 than those delivered in the first stage (14.1% vs. 3.6%). Admission to the NICU was more common among neonates delivered by second-stage cesarean Sect.  (32.9% vs. 13.5%).

### Factors associated with adverse maternal and perinatal Outcomes

Multivariable logistic regression analyses were performed to identify factors associated with maternal and perinatal adverse outcomes among pregnant women who undergo cesarean delivery at HUCSH (Table 4). In a fully adjusted model, second-stage cesarean delivery had more than four- and three-times higher odds of developing adverse maternal (AOR = 4.20, 95% CI: 1.95–9.05) and perinatal (AOR = 3.05, 95% CI: 1.65–5.65) outcomes, respectively, when compared with first-stage cesarean section. Similarly, women living in a rural area had nearly two-fold higher odds of maternal adverse outcomes compared with women from an urban area (AOR = 2.15, 95% CI: 1.10–4.52). In addition, birth weight ≥ 4000 g was associated with two-fold higher odds of adverse perinatal outcomes (AOR = 2.25, 95% CI: 1.20–4.22) compared with birth weight < 4000 g.

**Table 4:** Factors associated with adverse maternal and perinatal outcomes at Hawassa University Comprehensive Specialized Hospital

Variable	COR (95% CI)	AOR (95% CI)	*p*-value
Adverse maternal outcome			
Stage of cesarean delivery			
First-stage (1)	1	1	
Second-stage	**6.88 (3.78–12.53)**	**4.20 (1.95–9.05)**	**< 0.001**
Residence			
Urban (1)	1	1	
Rural	**3.41 (1.82–6.39)**	**2.15 (1.10–4.52)**	**0.043**
Gravidity			
Primigravida (1)	1	1	
Multigravida	1.09 (0.60–1.98)	1.12 (0.54–2.31)	0.760
Onset of labor			
Spontaneous (1)	1	1	
Induced	1.16 (0.62–2.18)	1.18 (0.56–2.49)	0.660
Adverse perinatal outcome			
Stage of cesarean delivery			
First-stage (1)	1	1	
Second-stage	**3.86 (2.22–6.71)**	**3.05 (1.65–5.65)**	**< 0.001**
Birth weight			
<4000 g (1)	1	1	
≥4000 g	**2.22 (1.05–4.68)**	**2.25 (1.20–4.22)**	**0.011**
Residence			
Urban (1)	1	1	
Rural	2.00 (1.12–3.58)	1.58 (0.82–3.04)	0.170
Gravidity			
Primigravida (1)	1	1	
Multigravida	1.02 (0.58–1.81)	1.08 (0.55–2.13)	0.820
Onset of labor			
Spontaneous (1)	1	1	
Induced	1.19 (0.66–2.13)	1.21 (0.61–2.41)	0.580

*AOR* Adjusted odds ratio, *COR* crude odds ratio

Bold value indicates statistical significance (*p* < 0.05)

## Discussion

This study assessed adverse maternal and perinatal outcomes of cesarean section performed during the first and second stages of labor at HUCSH, Southern Ethiopia. Cesarean delivery performed in the second stage of labor was associated with an increased risk of maternal and neonatal adverse outcomes compared with cesarean delivery performed at the first stage of labor. In addition, maternal residence in a rural area contributed to adverse maternal outcomes, whereas fetal macrosomia was associated with adverse perinatal outcomes independent of the stage of labor at which the CS was performed.

The findings in the present study were consistent with Ethiopian studies conducted in various regions, including Wolkite, Jimma, and Addis Ababa [8, 14, 17], as well as with other studies that reported similar results in Sudan, Kenya, Turkey, and India [7, 18–20]. The increased maternal intraoperative complications during the second stage of cesarean section, such as a higher rate of uterine incision extension and longer operation time, are related to the anatomical and technical challenges specific to this stage of labor [21, 22]. In this study, the rate of uterine incision extension and operation time of more than 50 min was more common in second-stage cesarean delivery than in first-stage cesarean section. This finding is supported by previous studies conducted in Nigeria and India [23, 24]. In addition, second-stage cesarean delivery was associated with a higher risk of uterine atony and postpartum hemorrhage, possibly due to longer myometrial contraction during labor, oxytocin receptor desensitization, and uterine muscle fatigue [25]. These findings were consistent with a study conducted in the United Kingdom and India [26, 27]. Therefore, it is crucial to consider effective prevention and management of intraoperative complications in second-stage cesarean section. In contrast, a study conducted in Israel reported that there was no association between second-stage cesarean section and uterine atony or blood loss of more than 1000 ml [28, 29]. Variations in study design, data collection methods, and disparities in obstetric care standards across the study settings may explain these discrepancies.

In this study, the second stage of labor was significantly associated with adverse perinatal outcomes, including a fifth-minute Apgar score < 7, and the need for NICU admission. The physiological stress of prolonged second-stage labor, compounded by potential fetal compromise before delivery and the technical difficulty of extracting a deeply engaged fetal head, likely contributes to these adverse neonatal outcomes. Similar results were reported in studies conducted in Wolkite, Eastern India, Kenya, and in a meta-analysis of ten cross-sectional studies conducted in 2014 [14, 19, 30, 31]. However, a study conducted in the United Kingdom shows that the adverse perinatal outcomes were higher in the first stage cesarean section than in the second stage cesarean delivery [26]. This difference may be explained by variation in the indications for cesarean section and the condition of the fetus before surgery.

Although neonatal mortality did not differ significantly between the two stages of cesarean delivery in our study, studies conducted in different countries show that neonatal mortality was significantly associated with second-stage cesarean delivery compared to first-stage cesarean Sects.  [19, 23, 26, 30]. The small number of neonatal deaths in our study may explain these discrepancies.

This study supports previous evidence of higher maternal and perinatal adverse outcomes in second-stage cesarean deliveries than in first-stage cesarean deliveries. Therefore, reducing rates of CS in the second stage of labor must be sought as the primary mode of prevention of adverse outcomes. To achieve this, it is recommended to direct efforts toward early detection of labor dystocia, making timely decisions before full cervical dilation, and providing a robust referral system for women in rural areas. The effectiveness of the second stage of cesarean deliveries can be enhanced by improving physicians’ expertise, ensuring preparedness for intraoperative complications, and strengthening immediate neonatal resuscitation [14, 31].

### Strengths and limitations

The strength of this study is that it uses a comparative design to evaluate adverse maternal and perinatal outcomes in first- and second-stage cesarean deliveries and includes an adequate sample, with oversampling of second-stage cases, to enable meaningful comparisons. However, a single-center, hospital-based design limits the generalizability of the findings to the wider community, and the cross-sectional design limits causal inference. Differences in indications between groups may limit comparability and introduce confounding. In addition, some relevant unmeasured factors, such as surgeon experience and detailed intraoperative technical variables, were not assessed.

## Conclusion

Cesarean section performed during the second stage of labor was significantly associated with adverse maternal and perinatal outcomes at HUCSH. Second-stage cesarean delivery increased the risk of low Apgar score, NICU admission, intraoperative complications, and postoperative morbidities. During the second stage of cesarean delivery, the obstetric team’s preparedness for potential intraoperative complications and immediate neonatal resuscitation should be strengthened.

## Data Availability

The datasets used during the current study are available from the corresponding author on reasonable request.

## Abbreviations

ANC: Antenatal care
APGAR: Appearance Pulse rate, Grimace Activity, and Reflex
CS: Cesarean Section
CPD: Cephalo-pelvic disproportion
EDHS: Ethiopia demographic and health survey
END: Early neonatal death
HUCSH: Hawassa University Comprehensive Specialized Hospital
IUFD: Intra-uterine fetal death
LMIC: Low- and middle-income countries
NICU: Neonatal intensive care unit
NRFHRP: Non-reassuring fetal heart rate pattern
PPH: Postpartum hemorrhage
WHO: World Health Organization
